# Endocannabinoid System Changes throughout Life: Implications and Therapeutic Potential for Autism, ADHD, and Alzheimer’s Disease

**DOI:** 10.3390/brainsci14060592

**Published:** 2024-06-10

**Authors:** Kamila Gabrieli Dallabrida, Joyce Maria de Oliveira Bender, Ellen Schavarski Chade, Nathalia Rodrigues, Tuane Bazanella Sampaio

**Affiliations:** 1 Department of Pharmacy, State University of Centro Oeste, Guarapuava 85040-167, PR, Brazil; 2 Department of Medicine, State University of Centro Oeste, Guarapuava 85040-167, PR, Brazil

**Keywords:** cannabidiol, THC, neurodegenerative diseases, neurodevelopmental disorders

## Abstract

The endocannabinoid system has been linked to various physiological and pathological processes, because it plays a neuromodulator role in the central nervous system. In this sense, cannabinoids have been used off-label for neurodevelopmental disorders, such as autism spectrum disorder (ASD) and attention-deficit/hyperactivity disorder (ADHA), as well as in Alzheimer’s disease (AD), a more prevalent neurodegenerative disease. Thus, this study aims, through a comprehensive literature review, to arrive at a better understanding of the impact of cannabinoids in the therapeutic treatment of patients with ASD, ADHD, and Alzheimer’s disease (AD). Overall, cannabis products rich in CBD displayed a higher therapeutic potential for ASD children, while cannabis products rich in THC have been tested more for AD therapy. For ADHD, the clinical studies are incipient and inconclusive, but promising. In general, the main limitations of the clinical studies are the lack of standardization of the cannabis-based products consumed by the participants, a lack of scientific rigor, and the small number of participants.

## 1. Introduction

Cannabinoids are compounds that act on cannabinoid receptors and can be categorized into three types: phytocannabinoids, which are derived from plants like cannabidiol (CBD); endocannabinoids, which are endogenous compounds, namely anandamide (AEA) and 2-arachidonoylglycerol (2-AG); and synthetic cannabinoids, such as dronabinol and nabilone [1].

The cannabis plant contains about 60 to 100 different cannabinoids. Phytocannabinoid research started in the late 19th century, which led to the isolation of cannabinol and CBD, as well as attempts to isolate delta-9-tetrahydrocannabinol (THC) in the 1940s. In the 1960s and 1970s, technological advances allowed the isolation and synthesis of several plant cannabinoids, including THC and CBD, leading to extensive pharmacological and physiological investigations. In the 1980s, the identification of cannabinoid receptor types 1 (CB1R) and 2 (CB2R), along with the discovery of endogenous agonists of these receptors, AEA and 2-AG, established the endocannabinoid system (ECS) [2].

Since then, the ECS has been linked to various physiological and pathological processes. The potency of cannabis is mainly determined by the THC concentration, its primary psychoactive component. THC is a CBR agonist with desirable effects, but also has addictive potential. On the other hand, CBD appears to have distinct properties, including opposite effects to THC [1]. CBD is a non-intoxicating ingredient, with preclinical evidence suggesting several biological proprieties, such as anti-inflammatory, neuroprotective, antipsychotic, analgesic, anticonvulsant, antiemetic, and antioxidant properties [3,4]. CBD acts in regard to several receptors and molecular targets, showing therapeutic properties throughout neuropsychiatric disorders, stemming from diverse central nervous system actions, including the agonist effect on the serotonin receptor (5-HT1A) associated with its anxiolytic and antidepressant properties [3]. In addition to this, adequate levels of both THC and CBD seem to have protective effects against neurological diseases [1].

Neurological disorders are a significant global public health issue due to their high prevalence, associated chronic disability, and severe impact on patients’ quality of life. Although the pathophysiology of these diseases is complex and not entirely understood, genetic, biological, and environmental factors are relevant to their appearance and progression [1,5]. There is increasing evidence of cannabinoids’ therapeutic potential on the behavioral impairments exhibited in neurodevelopmental and neurodegenerative disorders [5].

Once the ECS plays a neuromodulator role in the central nervous system, ECS stimulation could influence neurological conditions, even if caused by changes in other neurotransmitter systems. In this way, CB1R agonists have been studied, due to their ability to modulate neuronal activity and neurotransmitter release [5]. CBD and CB2R agonists are also promising strategies for avoiding the psychoactive effects associated with CB1R stimulation. The ECS plays a fundamental role in synaptic responsiveness and plasticity, affecting learning, memory, and executive functions [6].

Although the relevance of cannabinoids as a therapeutic alternative is notable, their usefulness in many neurological diseases remains unclear. Additionally, cannabis is now legal in many countries and is more accessible. Thus, this study aims, through a comprehensive literature review, to arrive at a better understanding of the impact of cannabinoids in the therapeutic treatment of patients with autism spectrum disorder (ASD), attention-deficit/hyperactivity disorder (ADHD), and Alzheimer’s disease (AD).

## 2. The Endocannabinoid System Changes throughout Life

The ECS is composed of cannabinoid G-protein-coupled receptors (GPCRs), which are part of the rhodopsin-like subfamily, with their endogenous ligands, and enzymes involved in the synthesis and degradation of these ligands. AEA and 2-AG are the main lipophilic chemicals of endocannabinoids, which act in a retrograde manner, being produced on demand. In summary, 2-AG is primarily synthesized by diacylglycerol lipase α and β (DAGL-α, DAGL-β) from diacylglycerol (DAG) and is mainly degraded by monoacylglycerol lipase (MAGL). The serine hydrolases, ABHD6 and ABHD12, also degrade 2-AG. In turn, AEA is primarily synthesized by phospholipase C (PLC) and N-acyl phosphatidylethanolamine phospholipase D (NAPE-PLD) from N-acyl phosphatidylethanolamine (NAPE) and is mainly degraded by fatty acid amide hydrolase (FAAH). Of note, 2-AG acts as a full cannabinoid receptor agonist, whereas AEA is a partial cannabinoid receptor agonist of cannabinoid receptors expressed in presynaptic neurons, which regulate the release of the other neurotransmitter. Nevertheless, cannabinoid receptors can bind with multiple ligands, which stabilize active receptor conformations and induce different intracellular pathways; this process is known as biased agonism [7].

CB1R has a large distribution in the mammalian brain, with significant expression in the basal ganglia, cerebellum, and hippocampus. However, it is scarce in the brainstem. Additionally, CB1R is present in the spinal cord, peripheral nerve terminals, cardiovascular system, gastrointestinal tract, and liver. As a result, these receptors modulate several neuronal functions, such as mood, appetite, and pain signaling [7]. Also, CB1R expression in different tissues, including the brain, liver, pancreas, skeletal muscle, and adipose tissue, is evidence of its role in energy homeostasis and, consequently, in feeding behavior and energy substrate accumulation [8]. On the other hand, CB2R is mainly expressed in the peripheral tissues. CB2R is primarily present in macrophage cells, including microglia, and in smaller quantities in other body parts, such as the reproductive, cardiovascular, gastrointestinal, and central nervous systems [7].

Some orphan GPCRs are also related to the ECS, such as GPR18, GPR55, and GPR119. Although these receptors share low sequence homology with CB1R and CB2R, they are modulated by various natural and synthetic cannabinoids [9]. GPR18 is highly expressed in the spleen, thymus, peripheral blood leukocytes, lymph nodes, cerebellum, lung, and testis. The modulation of GPR18 is associated with several biological processes, including pain, sperm physiology, immunomodulation, metabolism, and cancer [10]. On the other hand, GPR55 is highly expressed in dorsal root ganglion neurons and is activated by endocannabinoids and lysophosphatidylinositol [11,12]. It plays a significant role in several processes, including endothelium-dependent vasodilation, cellular proliferation and migration, inflammation, and pain management [12]. Notably, preclinical studies suggest that GPR55 reduces inflammatory and neuropathic pain and mitigates impaired adult hippocampal neurogenesis following chronic systemic inflammation [13,14]. Finally, GPR119 can be activated by the endocannabinoids’ oleoylethanolamine (OEA) and 2-AG [9]. This receptor is predominantly expressed in the gastrointestinal system and regulates energy balance in the pancreas, suggesting a putative role in type 2 diabetes, metabolic disorders, and obesity [15].

Interestingly, the constituent levels of the ECS may alter during aging, validating them as putative pharmacological targets. Aging can be defined as the process of becoming older, occurring from birth to death, or as the progressive physiological changes that induce biological function decline or senescence. In this sense, aging is the primary risk factor for the development of cognitive decline and neurodegenerative diseases. However, cognitive decline occurs even without a neurodegenerative disease diagnosis [16].

Cannabinoid receptors are heterogeneously distributed in fetal, neonatal, and adult human brains [17]. The fetal brain exhibits markedly higher densities of cannabinoid receptors in the basal ganglia compared to the neonatal brain. In particular, the globus pallidus internus in fetuses shows more than twice the cannabinoid receptor density found in neonates. Additionally, the midbrain region in neonates has substantially higher levels of cannabinoid receptors compared to adults, with significant increases observed in areas such as the substantia nigra pars reticulata, red nucleus, central gray, and superior colliculus [17]. Functionally, cannabinoid receptor binding is detected in the fetal period in the midbrain, brainstem, and cerebral cortex, suggesting an atypical and transient expression in the fetal brain compared to the adult brain [18]. These findings highlight the dynamic changes in cannabinoid receptor distribution during brain development [17,18].

Also, corticolimbic endocannabinoid signals change dynamically during early life and old age. The highest level of 2-AG is found in the fetal brain and near birth, with levels up to twice more than other ages [19]. During adolescence, the 2-AG levels fluctuate due to modifications in its synthesis, DAGLs, hydrolyzing enzymes, MAGL, and ABHD6 [20]. Of note, 2-AG synthesis is also catalyzed by DAGL-β in postnatal life, having had its expression reduced very rapidly in early life [21]. DAGL-α mRNA expression peaks between adolescence and young adulthood, MAGL gene levels are highest in childhood, and both enzymes are reduced during aging. ABHD6 expression increases continuously throughout life [20].

In turn, AEA levels are low in the fetal brain and increase gradually during childhood, with a peak in adolescence and a return to early life levels in adulthood [19,22]. Indeed, there is a significant increase in the mRNA expression of AEA synthesizing and hydrolyzing enzymes, NAPE-PLD and FAAH, respectively, throughout life. This continuous stimulation of AEA synthesis and degradation suggests the ongoing regulation of endocannabinoid signaling in adulthood [20]. Specifically, NAPE-PLD nearly doubles its gene expression from neonates to adulthood [20,23], while the increase in FAAH mRNA expression occurs to a lesser extent than the synthesis enzyme [20]. Importantly, FAAH activity fluctuates inversely with AEA during adolescence, supporting the AEA peak in this life phase [24]. Moreover, FAAH is colocalized with CB1R in many areas, except for the globus pallidus and substantia nigra pars reticulata, indicating major regulation of the formation of AEA [25].

CB1R expression peaks in early life, mainly in the striatum and prefrontal cortex, and stabilizes in adulthood, reaching a reduction of approximately 50% in CB1R mRNA levels and cannabinoid agonist binding in old age [20,26]. Of note, the decreased expression and function of CB1R begins and is more pronounced in limbic and associative regions, such as the medial prefrontal cortex. In contrast, CB1R expression reduction in sensorimotor cortices occurs only after adolescence [26]. The CB1R expression decrease coincides with cognitive maturation, indicating a possible attenuation of control over synaptic neurotransmission mediated by this receptor [20]. Of note, the CB2R expression profile throughout life remains unclear, but CB2R knockout mice display accelerated aging [27].

Moreover, sex differences were observed, with CB1R expression levels peaking earlier in females than in males [28]. The ECS and the female reproductive system are closely interconnected; a delicate balance between endocannabinoid production and CBR activity is necessary for the optimal functioning of the reproductive tract and hypothalamic–pituitary–ovarian axis. Specifically, the ECS activity and cerebral CB1R density fluctuate throughout the estrous and menstrual cycle, with the CB1R density being the highest during diestrus in the mediobasal hypothalamus of female rats and their affinity to cannabinoids is at its maximum during diestrus in the limbic forebrain [29]. Furthermore, plasma AEA levels are associated with serum FSH, LH, and E2 levels in premenopausal women and vary throughout the menstrual cycle. There is a plasma AEA peak at the time of ovulation, suggesting a possible association between the ECS and folliculogenesis and/or ovulation [30].

Overall, elevated CB1R and 2-AG degrading enzyme expressions in early life suggest a more refined form of control over this endocannabinoid. Indeed, the synthesizing enzyme DAGL-α begins to decline just in adulthood, whereas the presynaptic degrading enzyme MAGL is reduced from adolescence. However, ABHD6 expression, a postsynaptic degrading enzyme, increases during life, indicating anatomic control of the 2-AG levels in adolescence. As a result, the release of 2-AG from the postsynaptic terminal is reduced by the decline in DAGL-α and the increase in ABHD6 in adulthood, linking it to the maturation process [20]. In the same way, the FAAH elevation from adolescence culminates in major control of the AEA availability for CB1R modulation [24]. Thus, the suppression displayed by the ECS on presynaptic neurotransmission reduces after adolescence. 

These changes during development can contribute to the corticolimbic circuit neurons’ maturation, impacting inhibitory and excitatory neurotransmission in regard to the prefrontal cortex and establishing adaptative and complex cognition and behavior. Consequently, ECS signaling helps to enhance synaptic plasticity, enabling adolescents to incorporate new experiences and respond adaptively to the environment. This flexibility facilitates the acquisition of skills for independence and social interactions. However, disruptions in the ECS may impair the refinement of cortical circuits, affecting emotional behavior and the response to stress in the long term [31]. Indeed, it is possible that the age-dependent deteriorating brain, which is the basis of the neurodegenerative process, is linked, at least in parts, to ECS changes [32].

In this context, we hypothesize that the changes in ECS components could explain the therapeutic responses to cannabinoid products (natural or synthetic) observed in patients in different phases of life. Due to this, the therapeutic use of cannabinoids for neurodevelopmental disorders, such as ASD and ADHD, and the more prevalent neurodegenerative disease, AD, was revised.

## 3. Autism Spectrum Disorder

ASD is a multifactorial neurodevelopmental disturbance, defined by significant impairments in communication and social interaction. In addition, the core symptoms requested for ASD diagnosis include repetitive patterns of behavior and restricted interests that vary in severity. ASD is divided into three degrees according to the required support: level 1, requiring support; level 2, requiring substantial support; and level 3, requiring very substantial support [33]. In addition to the core symptoms, ASD carriers frequently have several comorbidities that directly impact their daily life. In general, patients with intellectual disability display behavioral disturbances, such as self-injury and irritability/aggression. However, ASD individuals with higher cognitive capacity are more affected by psychiatric diseases, i.e., anxiety, depression, and sleep problems. Also, epilepsy affects 5 to 30% of people with ASD, representing the most frequent comorbidity [34].

Although ASD is one of the most severe childhood disorders and has a high prevalence, ranging from 0.08 to 9.3% in the world [33], no effective treatments are currently available for the core symptoms. In this way, the therapeutic approach is focused on reducing disruptive behavior and skills training for greater independence. At this time the ASD etiopathogenesis remains unclear, and the pharmacotherapy is also limited, being restricted to antipsychotics, antiepileptics, selective serotonin reuptake inhibitors, stimulants, and anxiolytics for the treatment of comorbidities [34].

Among other neurotransmission systems, i.e., the serotoninergic and GABAergic systems, preclinical and clinical studies have demonstrated an imbalance in the ECS in ASD [35,36,37,38]. Children diagnosed with ASD show lower AEA plasma levels, as well as the related endogenous compounds N-palmitoylethanolamine and N-OEA, than those found in controls [35,36]. Indeed, the increased AEA levels induced by FAAH inhibition improve social impairments through CB1R modulation, while PPARα, PPARγ, and GPR55 expression in the frontal cortex and hippocampus were reduced in rodent models of ASD [37,38]. Also, the ECS regulates social interaction, the emotional response, and behavioral reactivity to a particular context, functions usually disrupted in ASD [34].

In this way, exogenous cannabinoids, like phytocannabinoids, emerge as putative therapeutic approaches for ASD symptoms (Table 1). Acute CBD beneficial effects were demonstrated in rodent ASD models [39,40]. CBD-treated BTBR mice enhanced their social interaction preference, attenuated social novelty preference, and anxiety-like behavior [39,41]. A prosocial improvement was also observed in mice exposed only to a terpene blend from CBD oil [41]. Additionally, CBD improved the performance of *Fmr1-^Δ^exon 8* rats in a novel object recognition task without causing tolerance, even after repeated administration. The mnemonic effects of CBD were blocked by the hippocampal administration of a GPR55 antagonist, suggesting the involvement of this endocannabinoid receptor. The study also revealed a decrease in FAAH expression in the control group, which was not observed in *Fmr1-^Δ^exon 8* rats [40]. However, Poleg et al. [42] reported that isolated CBD or CBD-rich oils did not improve social deficits. In contrast, THC oil enhanced social deficits, repetitive grooming, and anxiety-like behaviors by over 70% compared to non-treated *Shank3* mutant mice. This effect was associated with the activation of CB1R [42].

In this context, Kurz and Blaas [43] reported for the first time on the use of THC by a child of six years old with autistic disorder, with beneficial effects on hyperactivity, irritability, lethargy, stereotype, and inappropriate speech. A retrospective case series containing 21 ASD patients treated with different sublingual cannabis extracts (equivalent CBD and THC, or a level high for each one) for at least three months, demonstrated an improvement in at least one of the core ASD symptoms and comorbidities, such as sensory difficulties, sleep disorders, and/or seizures. Importantly, the adverse effects observed were solved through strain change [44].

Corroborating with these findings, the therapeutic effects of cannabis extracts on ASD carriers were also described in other case reports, without reported side effects [52,55,56]. Ponton, Smyth, Soumbasis, Llanos, Lewis, Meerholz, and Tanguay [52] relayed that a CBD-rich extract at very low doses (4 mg CBD and 0.2 mg THC, twice a day) improved behavioral symptoms, such as anxiety, sleep, and social deficits, in a 15-year-old boy. A nine-year-old male patient treated with a full-spectrum high-CBD and low-THC oil showed a reduction in violent outbursts, self-injurious behaviors, and sleep disruptions, as well as an improvement in social interactions, concentration, and emotional stability [55]. In contrast, a 17-year-old teenager displayed a decrease in aggressiveness after treatment with a terpene-enriched CBD oil, but not with CBD oil alone, suggesting that the addition of selected and safe terpenes increases the efficacy of CBD therapy [56].

Based on these findings, it is plausible to consider that both CBD and THC alone seem to present low therapeutic effects on ASD. Indeed, a randomized, double-blind, placebo-controlled trial revealed that pure CBD or THC did not demonstrate any effect significantly different from the placebo [53]. Nevertheless, the participants who received a CBD/THC extract at a ratio of 20:1 showed a significant improvement in their disruptive behavior and social responsiveness score compared to the placebo [53,59], without an improvement in sleep disturbances [59]. Furthermore, in a retrospective cohort treated with a full-spectrum CBD-rich cannabis extract, Montagner et al [60] observed that 18 out of 20 patients showed an improvement in their core and comorbidity ASD symptoms, such as communication, social interaction deficits, and sleep disorders, impacting positively on the quality of life of the patients and their families.

Other observational studies using CBD/THC extracts at a ratio of 20:1 reinforce the therapeutic potential of cannabinoids in ASD. Most patients were treated with cannabis oil containing 30% CBD and 1.5% THC, three times a day for six months, reaching an average daily dosage of 79.5 ± 61.5 mg CBD and 4.0 ± 3.0 mg THC. ASD patients relayed consistent improvements in their quality of life, mood, seizure occurrence, ability to dress and shower independently, sleep, and concentration [47]. CBD-rich extracts also act on core ASD symptoms, ameliorating rage attacks, agitation, and speech impairment [45,46,47]. A CBD-rich cannabis treatment for 6 months in children and adolescents with ASD improved their social communication abilities as quantified by the Autism Diagnostic Observation Schedule 2 (ADOS-2), the Social Responsiveness Scale (SRS), and the Vineland Adaptive Behavior Scales. Participants with more severe initial symptoms showed larger improvements following the intervention, independent of age or final cannabis dosage [57]. Of note, the prospective phase III study demonstrated a positive safety profile of CBD/THC oil at a ratio of 20:1, because no clinically significant differences in the plasma parameters were found in children with ASD [58].

A CBD content increase at a ratio of 75:1 CBD/THC (mean daily dosage: CBD 175 mg/day (100–350) and THC: 2.33 mg/day (1.33–2.33)) during 6 to 9 months of treatment induced strong improvements in seizures, ADHD, sleep, communication, and social interaction [48]. In addition, Efron et al. [54] carried out a phase I/II double-blind, parallel-group, randomized, placebo-controlled pilot study to investigate whether CBD reduces several behavioral problems in children and adolescents with intellectual disability. CBD oil at a maintenance dose of 20 mg/kg/day was effective in reducing severe behavioral problems. Moreover, the medication was generally well tolerated, without the need for dose reduction adjustments due to adverse events, serious adverse events, or clinically significant abnormal laboratory test results [54].

Interestingly, acute CBD effects in ASD subjects were investigated in randomized placebo-controlled clinical trials using magnetic resonance spectroscopy and functional magnetic resonance imaging [49,51]. It was found that CBD alters glutamate and GABA systems both in ASD and typically developing controls, but GABA transmission in the prefrontal cortex is differently affected in ASD patients [49]. In contrast, cannabidivarin (CBDV), another cannabis derivative, also administrated in an acute manner, modulates glutamate/GABA systems in the basal ganglia, but not in frontal areas, of ASD carriers [50]. Lastly, CBD was able to alter the fractional amplitude of low-frequency fluctuations in the cerebellar vermis, as well as its functional connectivity with several subcortical (striatal) and cortical targets [51]. Moreover, cannabis-responsive lipid-based biomarkers in saliva samples from children with ASD undergoing treatment with cannabis extracts were identified, suggesting that these lipid metabolites may potentially serve as indicators of response to cannabinoid treatment [61].

Of note, cannabis appears to be well tolerated and safe in individuals with ASD, with adverse effects being mild and/or transient. The side effects most reported included restlessness, somnolence, irritability, dizziness, change in appetite, and unexplained laughter [45,46,47,48,58,62].

Overall, the findings seem promising, mainly for the use of cannabis extracts rich in CBD during childhood. Of special interest, none of the revised studies focused on correlating the effects experienced by the participants with their age. However, most of the population included in the clinical studies had a similar average age (defined as childhood, between 5 and 12 years old) [45,46,47,48,53,57,58,59,61,62], who are expected to have elevated AEA and CB1R activity levels [19,20,22,26]. Importantly, ASD children display reduced plasma AEA levels [35,36]. Although CBD is a non-competitive negative allosteric modulator of CB1R and a low-affinity agonist of CB2R, other pharmacological targets seem to mediate the therapeutic effects of CBD. In this context, the beneficial effects of CBD for ASD children could be related to its inhibitory action on FAAH activity, resulting in increased AEA levels, and agonism in relation to TRPV1 and 5-HT1A receptors that mediate anti-convulsant and behavioral functions [1,3]. However, large, randomized, placebo-controlled clinical trials are necessary to fully understand the benefits and risks of CBD-rich extract use by different age groups, mainly children and adults. Finally, the mechanism of action of CBD in ASD needs to be better characterized.

## 4. Attention-Deficit/Hyperactivity Disorder

ADHD is a neurodevelopmental disorder, with a prevalence range from 0.1 to 8.1% in children and adolescents, and between 0.6 to 7.3% in adults [63]. It is characterized by three groups of manifestation: impulsivity, inattention, and hyperactivity. Patients diagnosed with ADHD may experience a variety of symptoms that mainly involve executive functioning, impairing the ability to organize, plan, manage time, maintain, or change focus, and remember details. Also, ADHD children and adolescents often experience mood dysregulation and difficulties with social and academic functioning. These symptoms can be present at all stages of life [64].

Regarding its pathophysiology, it is known that ADHD individuals have a dysfunction in dopaminergic release in various regions of the brain, including the frontal, subcortical, and limbic regions. Additionally, magnetic resonance imaging studies have demonstrated a reduction in neural activity in the frontal region, anterior singular cortex, and basal ganglia in ADHD patients [64]. These changes directly affect the release of neurotransmitters, such as dopamine and norepinephrine. Of special interest, the ECS has been linked to dopamine production, suggesting a connection between endocannabinoids and ADHD [65].

According to what has been previously mentioned, changes in different components of the ECS can contribute to a variety of neurological disorders, primarily due to differences in the gene and protein expression of CB1R. Indeed, a high concentration of CB1R is found in areas associated with cognitive processing and functioning; it also plays a crucial role in regulating the release of monoamines [65]. Importantly, decreased CB1R gene and protein expression was found in the brainstem [66]. In addition to this, CB1R agonism stimulates an anxiolytic-like effect and exhibited an increase in locomotion in an animal model of ADHD [67]. Moreover, AEA degradation seems impaired in ADHD children, affecting neurotransmitter release and brain plasticity [68].

ADHD treatment typically consists of a combination of stimulant and non-stimulant drugs, together with cognitive behavioral therapy. Psychostimulants are the first-line pharmacotherapy, being considered the most effective therapy. However, these drugs can cause side effects, such as insomnia and reduced appetite, resulting in low adherence rates among ADHD patients [64]. Thus, there is an urgent need to explore alternative therapeutic approaches to treat ADHD.

In this context, the use of cannabis has been suggested as an alternative therapy for ADHD. Although its relevance as a therapeutic alternative is notable, the literature examining the effects of cannabis on ADHD symptoms is scarce and ambiguous. Preclinical studies have examined the therapeutic potential of cannabis in regard to behavioral tasks related to ADHD symptoms, however they did not use animal models of ADHD. In contrast, the off-label use of cannabis in ADHD provides clinical and anecdotal evidence, suggesting that the therapeutic benefits of cannabis for this disorder are growing.

In this sense, a series of case reports present positive results from using cannabis-based medications to treat ADHD patients. Mansell et al. [69] described the cases of three male patients, aged 18, 22, and 23 years old, who began self-medicating with cannabis. They used a combination of cannabis oil containing 20:1 CBD/THC and THC-rich cigarettes, alongside medication previously prescribed by their psychiatrist. Comparing the periods before and after the cannabis treatment, the patients showed improved performance in standard scales used to assess depression and anxiety levels, emotional regulation, and attention deficit. The ADHD patients also related clear amelioration in regard to their focus, social skills, and emotional control, which improved their quality of life. In addition, the side effects experienced were mild and did not interfere with their day-to-day activities [69].

Similarly, a case study evaluated a 33-year-old Finnish male patient who used methylphenidate for six years and discontinued it due to its long-term adverse effects. Bedrocan^®^ (THC 20%; CBD 0.5%) and Bediol^®^ (THC 6.3%; CBD 8%) were prescribed. According to the medical report, the patient experienced reduced hyperactivity and impulsivity, and improved focus after using THC 20% and CBD 0.5% for five years. However, after this period, sleep problems and agitation occurred. To treat these symptoms, THC 6.3% and CBD 8% were prescribed, improving the quality of the patient’s sleep pattern [70].

A recent study reported the results of 68 ADHD patients (mean age = 35.62 ± 10.23 years old) who used medicinal cannabis products, including the oral use of cannabis oil and/or inhalation of dried flowers (mean dose for all patients: 15.0 mg/day CBD and 208.75 mg/day THC). The ADHD carriers were evaluated after 1, 3, and 6 months of treatment, resulting in significant improvements in the quality of life, generalized anxiety disorder, and sleep quality. Side effects, such as dry mouth, insomnia, and impaired concentration, were reported by only 11 participants. Additionally, there was a reduction in the concomitant use of medications for ADHD during the study [71].

Corroborating these findings, a large observational study (n = 1738 students) reported that 91.93% of participants reported improvements in their ADHD symptoms, such as hyperactivity, impulsivity, and restlessness, after starting cannabis treatment. Patients who used cannabis alongside other ADHD drugs reported a reduction in the adverse effects. Only 4.35% of patients experienced worsening symptoms, which were related to memory deficits, while 3.73% reported no effect [72].

Of note, a pilot clinical trial, randomized, triple-blind, and placebo-controlled study evaluated the effectiveness of Sativex^®^ (THC and CBD in equal proportions (1:1)) in 30 adults diagnosed with ADHD (mean age = 36.91 ± 11.70). The primary outcome measured the cognitive performance and activity level, while the secondary outcome included ADHD symptoms and emotional ability. There was no significant difference in the primary outcome. However, the use of 1:1 THC:CBD was associated with significant effects against hyperactivity and impulsivity [73].

To better understand the dose–response relationship of cannabinoids and terpenes on ADHD symptoms, 59 patients, aged 18 or older, were divided into two subgroups based on the used cannabinoid dosage. The scores on the Adult ADHD Self-Report Scale were lower, indicating fewer ADHD symptoms, for patients who used high monthly doses (17–41 mg) of cannabinoids. This group also showed lower anxiety scores compared to the subgroup taking low monthly doses of cannabinoids (12–20 mg). In addition, the subgroup using high doses of cannabinoids discontinued other ADHD drugs, while this did not happen in the low-dose subgroup. Additionally, the high-dose subgroup also consumed significantly higher amounts of the phytocannabinoids, THC, tetrahydrocannabivarin (THCV), CBD, cannabinol, cannabichromene, cannabigerol, tetrahydrocannabinol-C4, and the terpene trans-β-farnesene, which may contribute to the observed beneficial effects [74].

Based on the above, there is a set of evidence that supports the use of cannabis as a therapeutic agent for ADHD. However, these studies have some limitations, such as the small number of research subjects, study design, and lack of standardization of the dose of cannabinoids, which limits the relevance of the findings. Of note, these studies only evaluated adult patients aged 18 and over, which restricts the investigation of a possible difference in the efficacy of cannabinoid treatment for ADHD in different age groups, such as childhood and early adolescence.

Therefore, we emphasize that the need for continuous scientific research is noteworthy, particularly large randomized clinical trials, for the elucidation of the use of cannabis as an alternative treatment for ADHD. Moreover, both preclinical and clinical research fail to elucidate the mechanisms involved in the pharmacological potential of cannabinoids for ADHD.

## 5. Alzheimer’s Disease

Considered the most common neurodegenerative disorder, AD induces cognitive decline associated with the amyloid beta (Aβ) pathology. Although its etiology remains unknown, AD is classified into two forms: late-onset sporadic AD and early-onset familial AD. Familial AD comprises less than 5% of cases and is also known as a genetic form of the disease, resulting in autosomal dominant mutations in the presenilin 1 and 2 (PS1 and PS2) and amyloid precursor protein (APP) genes. In this way, APP is cleaved by γ- and β-secretases (which are encoded by PS1 and PS2) in regard to two forms of toxic Aβ proteins: Aβ_40_ and Aβ_42_. On the other hand, even if it is more prevalent, sporadic AD is less understood. There is evidence of a complex interaction between genetic susceptibility and environmental factors [6,75].

However, both AD types converge to the same disease progression. Classically, AD begins with mild cognitive impairment and progressively reaches dementia. During the early stages, deficits in short-term and spatial memory, communication, and learning are observed. Emotionality, eating, and dressing are affected in the intermediate phases. Lastly, cognitive disruption is reached in advanced stages, in which AD carriers need full-time care [6]. Similarly, the neuropathological hallmarks are also the same: aggregates and senile plaques composed of the Aβ protein and neurofibrillary tangles of the hyperphosphorylated tau protein. These processes induce oxidative stress and chronic inflammation, which potentiate the neurodegenerative cascade and cognitive decline [75].

AD pharmacotherapy only provides limited benefits associated with several adverse effects. In this sense, new therapeutic strategies have been evaluated both in preclinical and clinical trials. Among them, the major compounds of cannabis, CBD and THC, have attracted much attention. A summary of the preclinical and clinical evidence related to cannabis derivatives and AD is presented in Table 2.

The first evidence of CBD-positive action against Aβ toxicity was reported by Iuvone et al. [78]. CBD beneficially interferes with several Aβ-triggered neurodegenerative pathways [76,81,84]. The CBD pretreatment demonstrated neuroprotective, anti-apoptotic, and antioxidant actions in neuronal cells exposed to the Aβ peptide [77,78,83]. Following this, Esposito et al. [76] found that in vitro CBD inhibits tau hyperphosphorylation through the wingless-type MMTV integration site family member (Wnt)/β-catenin pathway, and induces APP ubiquitination and restores the hippocampal long-term potentiation (LTP) via peroxisome proliferator-activated receptor-γ (PPARγ) interaction [84,90]. CBD can also induce slight changes in the conformation of the tau protein, creating a stable tau–CBD complex and preventing tau aggregation [93]. In addition, the anti-inflammatory effect of CBD against Aβ toxicity involves microglia modulation, resulting in the suppression of proinflammatory mediators, for example, inducible nitric oxide synthase (iNOS) enzyme levels decrease via p38 MAP kinase phosphorylation and nuclear factor kappa B (NF-κB) [77,83]. Lastly, CBD downregulates the mRNA expression of kinases responsible for tau phosphorylation and β- and γ-secretase in mesenchymal stem cells [106], and modulates microglial cell function through CBR and the adenosine A_2A_ receptor [82].

In vivo, CBD effects were also investigated. Corroborating with the in vitro data, CBD showed an anti-inflammatory effect against the reactive gliosis and elevated proinflammatory markers, such as interleukin (IL)-1β, IL-6, TNF-α, PLCG2, and CTSC induced by Aβ injection in rodents [80,81,82]. PPARγ mediation on the CBD effects was also reinforced, including the stimulation of hippocampal neurogenesis. Furthermore, CBD causes a decrease in Aβ-enhanced microglial activation in some critical regions of the hippocampus, including dentate gyrus and CA2, and cortical areas [81]. Importantly, CBD-treated mice display improved learning performance in a spatial memory task disrupted by Aβ_1–40_ and Aβ_1–42_ [82]. In this way, cognitive deficits addressed in the social recognition and novel object recognition tasks were restored by CBD in AβPP/PS1 transgenic mice [85,86]. In contrast, CBD did not alter the anxiety-like, motor, and fear-conditioning parameters observed in TAU58/2 mice [92].

Current AD therapy involves cholinesterase activity inhibitors, and research is now exploring the potential of inhibitors of secretase enzymes in AD, especially related to β-secretase. Of note, Mooko et al. [94] revealed that several cannabis extracts had inhibitory potential on both cholinesterase and β-secretase activity in vitro, with low cytotoxicity. Specifically, hexane and dichloromethane (DCM) extracts showed superior inhibition of cholinesterase activity, while water, DCM/methanol (1:1), and methanol showed superior inhibition of β-secretase activity. CBD inhibited both acetyl- and butyrylcholinesterases, indicating that it could be a potential anticholinergic agent for the symptomatic treatment of AD [94].

Regarding THC, an inhibitor of acetylcholinesterase activity, Aβ aggregation, and microglial-activated neurotoxicity in vitro [79,83], a synergistic effect was observed with CBD [87,91]. THC+CBD treatment meliorates the learning in two behavioral cognitive tests, the Aβ pathology and neuroinflammation found in AβPP/PS1 mice in the early symptomatic phase of AD and the synergic treatment was more effective than CBD or THC treatments alone [87]. However, THC+CBD was effective only against the mnemonic deficit in AβPP/PS1 mice in AD advanced stages. Interestingly, although the CB2R deficiency in APP/PS1 mice exacerbates the Aβ pathology, its absence did not affect the cognitive effect induced by THC+CBD, suggesting a minor role for CB2R in this action [88]. Furthermore, THC+CBD effects seem to be linked to reduced GluR2/3, increased levels of GABA-A Rα1, and suppressed oxidative stress and inflammation [89,91].

Recently, acidic variants of CBD (CBDA) and THC (THCA) have also shown positive effects in both in vitro and in vivo assays. CBDA or THCA treatments significantly reduced primary neuronal cell death caused by exposure to Aβ_1–42_. Additionally, these treatments modulate Ca^2+^ homeostasis and avoid the increase in APP, polymeric Aβ, oligomeric Aβ, and p-tau levels induced by Aβ_1–42_. Further, BDNF, p-TrkB, and p-CREB levels were increased by CBDA and THCA. In female mice, CBDA or THCA protected spatial and object recognition memories disrupted by Aβ_1–42_ [95].

Even though the preclinical data is encouraging, clinical evidence reveals an unclear context for the use of cannabis derivatives in AD. In general, clinical data demonstrates that oral THC at a minimum dose of 2.5 mg/day may improve neuropsychiatric symptoms secondary to AD, mainly agitation, anorexia-associated weight loss, and nocturnal disturbances [96,97,98,99]. Additionally, Shelef et al. [102] and Palmieri and Vadala [105] reported similar effects with cannabis oil at different concentrations, including delusions, agitation/aggression, irritability, apathy, eating disturbances, sleep and caregiver distress, among the decreased domains in the Neuropsychiatric Inventory score (NPI). Moreover, cognitive improvement in the Mini-Mental State Examination (MMSE) questionnaire was observed in 45% of patients treated with different doses of cannabis oil containing 22% THC and 0.5% CBD [105]. Interestingly, in a case report, Ruver-Martins et al. [104] verified a cognitive improvement in the MMSE and Alzheimer’s Disease Assessment Scale (ADAS-Cog) that was stable for more than one year in a patient treated with microdoses of THC/CBD oil in a ratio of 8:1. These findings suggest that low doses (500 µg) of this cannabis extract can be effective in AD symptom suppression, without the presence of significant side effects or toxicity [104].

However, phase II clinical studies showed no significant differences in the NPI score, Cohen-Mansfield Agitation Inventory (CMAI), quality of life, and activities of daily living according to the Barthel Index between the placebo and oral THC (dose range from 1.5 to 4.5 mg/day) [100,101,103,107]. These data are corroborated by a meta-analysis study, which included six randomized, placebo-controlled trials involving AD carriers who received oral THC, and found no significant differences in aggression and agitation [108].

Importantly, oral THC treatment may be safe and well tolerated in AD patients, with euphoria, somnolence, and tiredness being the main adverse effects [100,101]. Moreover, the putative risk of falls due to changes in balance and gait was not confirmed. Alternatively, oral THC showed beneficial adverse effects on locomotion in AD patients [103].

Overall, preclinical evidence supports that cannabis components may be useful as novel therapeutic strategies for AD. However, the clinical data do not encourage this. It should be highlighted that in vitro and in vivo studies suggest that the synergistic effect of THC and CBD could be more effective than THC or CBD alone, and this approach has not been investigated in randomized, double-blind, placebo-controlled trials. Moreover, due to numerous preclinical evidence of the neuroprotective effects of CBD, clinical trials involving this compound could also reveal an interesting tool to manage AD. 

## 6. Final Considerations

Based on the above, it is noticeable that cannabinoid research in neurodevelopmental and neurodegenerative diseases is growing. A better understanding of ECS and the changes in its components throughout a person’s lifespan sheds light on the novel biological targets. Herein, we described that mainly CB1R, AEA, 2-AG, and its synthetic and metabolic pathways are modulated during childhood, adolescence, and old age. Of note, ECS components also are altered in some neurological disorders, such as ASD, ADHD, and AD. Importantly, cannabinoid replacement, through exogenous cannabis derivates, for example, CBD and THC, is promising for these diseases. Specifically, cannabis products rich in CBD displayed a higher potential in ASD children, while cannabis products rich in THC have been tested more for AD therapy. For ADHD, the clinical studies are incipient and inconclusive, but promising. In general, the main limitations of the clinical studies are a lack of standardization of the cannabis-based products consumed by the participants, a lack of scientific rigor, and the small number of participants. Of special interest, putative differences in the cannabinoid treatment effects on different age groups need to be tested, primarily for ASD and ADHD patients.

## Figures and Tables

**Table 1:** Preclinical and clinical evidence of the use of cannabinoids in ASD models and patients.

**Preclinical Studies**			
**Model**	**Treatment**	**Main Findings**	**References**
InsG3680 *Shank3* mutant mice	CBD/THC ratio 25:1 oil (25 mg/kg CBD, 1 mg/kg THC), CBD oil (25 mg/kg CBD), or THC oil (1 mg/kg THC) or olive oil (5 mL/kg) intragastric, twice a week, for 3 consecutive weeks.	THC oil is preferable for treating autistic-like phenotypes. THC oil improved social deficits, repetitive grooming, and anxiety-like behaviors. CBD/THC ratio 25:1 oil decreased the glutamate concentration in the CSF. These effects were linked to CBR1 activation.	[42]
BTBR *T^+^ Itpr3^tf^/J* (BTBR) mice	Inhaled CBD isolate oil. Specifically, 83.3 µL vapored for 6 s, every 5 min, totaling 30 min.	Inhaled CBD improves prosocial and anxiety-like behaviors. Only the terpene blend, also enhanced prosocial behavior, independently of CBD.	[41]
Adult male BTBR, C57BL/6J and SERT KO mice	CBD 0.1, 1, or 10 mg/kg, i.p., 30 min before beginning the tests.	CBD (10 mg/kg) enhanced social interaction preference and attenuated social novelty preference in BTBR mice. No behavioral difference was observed in C57BL/6J or SERT KO mice treated with CBD.	[39]
WT and *Fmr1-^Δ^exon 8* male rats	Acute treatment: CBD 10 mg/kg or vehicle, i.p., 2 h before testing, at PND40. Chronic treatment: CBD 10 mg/kg or vehicle, i.p., once daily for three weeks (PND 19 to PND39).	CBD reduced short-term object recognition deficits displayed by *Fmr1-^Δ^exon 8* rats, without inducing tolerance after repeated administration, through hippocampal GPR55 receptor stimulation.	[40]
**Clinical Studies**			
**Study Features**	**Treatment**	**Main Findings**	**References**
Case report of a child (6 years old)	THC (initial dosage of 0.62 mg, gradually increased to 3.62 mg).	THC enhanced hyperactivity, irritability, lethargy, stereotype, and inappropriate speech.	[43]
Retrospective case series (n = 21; mean age = 9 years old)	Sublingual cannabis extracts (71.5% received balanced CBD:THC extract, 19% high CBD, and 9.5% high THC extracts) for at least three months.	Cannabis extracts improved at least one of ASD’s core symptoms and comorbidities, such as sensory difficulties, sleep disorders, and/or seizures.	[44]
Observational study (n = 53; mean age = 11 years old)	CBD-rich oil at a daily dose of CBD 16 mg/kg (maximal daily dose 600 mg), and THC 0.8 mg/kg (maximal daily dose of 40 mg).	20:1 CBD/THC was associated with improvements in various comorbidity symptoms of ASD, such as hyperactivity, self-injury and rage attacks, sleep problems, and anxiety. These results were not statistically different from conventional treatments.	[45]
Retrospective study (n = 60; mean age = 11 years old)	20:1 CBD/THC extract, two or three times a day, starting with 1 mg/kg/day of CBD and a maximal dose of 10 mg/kg/day of CBD.	CBD-based treatment improved the behavioral outbreaks in 61% of ASD children. Side effects included sleep disorders (14%), irritability (9%), and loss of appetite (9%). One serious psychotic event was observed after a higher dose of THC.	[46]
Observational study (n = 188; mean age = 12 years old)	CBD-rich oil in a ratio of 20:1 (30% CBD and 1.5% THC).	CBD-rich oil is well tolerated, safe, and effective in relieving symptoms of ASD children, including seizures, tics, depression, restlessness, and rage attacks. Significant improvements were reported in the quality of life, mood, ability to perform daily activities, sleep, and concentration, after 6 months of treatment. Specifically, 34.3% of patients reduced the use of concomitant medications. Improvement in a child’s global assessment was reported by more than 80% of parents.	[47]
Observational study (n = 18; mean age = 10 years old)	Approximately 75:1 CBD/THC administered in capsules containing, respectively, 25 or 50 mg of CBD and 0.34 or 0.68 mg of THC.	Among the 15 patients who adhered to the treatment, only one patient failed to improve in regard to their ASD symptoms after 6–9 months of treatment. The strongest ameliorations were seizures, ADHD, sleep disorders, communication, and social interaction deficits.	[48]
Placebo-controlled, randomized, double-blind, repeated-measures, crossover case-control study (n = 34; mean age = 30 years old)	600 mg of CBD, or 600 mg of CBDV, or placebo.	CBD modulates glutamate/GABA systems, but the prefrontal GABA system is decreased in ASD. CBD significantly increased the fractional amplitude of low-frequency fluctuations in the cerebellar vermis and the right fusiform gyrus in the ASD group, with no significant change in the controls, and altered vermal functional connectivity with several of its subcortical and cortical targets in the ASD group. CBDV also significantly increased glutamate in the basal ganglia of ASD patients.	[49,50,51]
Case report of a teenager (15 years old)	0.1 mL of 20:1 CBD/THC (2 mg CBD and 0.1 mg THC), twice a day, with an increase of 0.1 mL per dose if no effects were observed, up to a maximum of 0.5 mL (10 mg CBD and 0.5 mg THC).	Low doses of CBD/THC (4 mg CBD and 0.2 mg THC) improved anxiety, sleep, and social deficits.	[52]
Randomized, double-blind, placebo-controlled trial (n = 150; mean age = 11 years old)	Oral placebo, CBD/THC at a 20:1 ratio (167 mg/mL CBD and 8.35 mg/mL THC), pure CBD and pure THC at the same concentration.	No significant differences in the total scores of the HSQ-ASD and the APSI among the treatment groups. Disruptive behavior, assessed by CGI-I, and median SRS total score showed a significant improvement in participants who received CBD/THC extract compared to the placebo.	[53]
Phase I/II double-blind, parallel-group, randomized, placebo-controlled pilot study (n = 8; mean age = 13 years old)	Starting dose of CBD at 5 mg/kg/day (two doses). The dose was increased in increments of 5 mg/kg every 3 days for 9 days up to the maintenance dose of 20 mg/kg/day. A maximal dose of 1000 mg/day was administered to all participants weighing 50 kg or greater.	CBD was effective in reducing severe behavioral problems in children and adolescents with intellectual disability.	[54]
Case report of a child (9-year-old)	High-CBD and low-THC oil (20 mg of CBD and <1 mg of THC/mL). The starting dose was 0.1ml, twice daily, increased every three to four days until 0.5 mL, twice daily.	High-CBD and low-THC oil treatment reduced the behavioral deficits, including violent outbursts, self-injurious behaviors, and sleep disruptions, and improved social interactions, concentration, and emotional stability, without side effects.	[55]
Case report of a teenager (17 years old)	Terpene-enriched CBD oil, three times/day, corresponding to 11.2 mg CBD/day and 0.19 mg/kg.	Terpene-enriched CBD oil was more effective than CBD oil alone in treating aggression associated with ASD.	[56]
Open-label study (n = 110; mean age = 9 years old)	CBD/THC oil 20:1, starting with one daily drop containing 5.7 mg CBD and 0.3 mg THC, with a gradual increase in dosage until parents perceived improvements.	Significant improvements were observed in ADOS-2, SRS, and Vineland scores after a 6-month treatment. Participants with more severe initial symptoms showed larger improvements following the intervention, regardless of age or final cannabis dosage.	[57]
Prospective cohort (n = 59; mean age = 10 years old)	CBD/THC oil 20:1, starting with one daily drop containing 5.7 mg CBD and 0.3 mg THC, with a gradual increase in dosage until parents perceived improvements.	No significant changes were observed in total blood count, urea, creatinine, liver enzymes, thyroid hormones, or other hormones after 3 months of treatment. LDH and TSH levels were significantly lower after 3 months of treatment compared to the baseline, while the free T4 levels were significantly higher. There were no significant differences in the biochemical parameters between the group receiving concomitant medications and the group receiving only the cannabis oil, except for a higher potassium level in the group not receiving additional medications. All values remained within the normal range.	[58]
Placebo-controlled trial (n = 150; mean age = 11 years old)	Whole-plant extract (CBD/THC ratio 20:1), pure cannabinoids (CBD/THC at a 20:1 ratio), and placebo. The starting dose was 1 and 0.05 mg/kg/day of CBD and THC, respectively, or placebo. The dose was increased every other day, up to 10 and 0.5 mg/kg/day of CBD and THC, respectively, for children weighing 20–40 kg; or 7.5 and 0.375 mg/kg/day of CBD and THC, respectively, for weight >40 kg until 420 mg CBD and 21 mg THC/day, divided into three daily doses.	CBD-rich cannabinoid treatment did not improve sleep disturbances more than the placebo, accessed by CSHQ. However, the total CSHQ scores were associated with improvements in the ASD core symptoms, as indicated by the SRS scores.	[59]
Retrospective cohort (n = 20; mean age = 14 years old)	Treatment starts with low to intermediate doses of a CBD-rich extract (25–50 mg CBD/day), which were slowly increased over 4 to 12 weeks between 50–150 mg CBD/day.	Overall, 18 out of 20 patients showed improvements in symptoms of ASD, such as behavioral disorders, communication, social interaction deficits, and sleep disorders, and in the quality of life for patients and their families. Side effects were mild and infrequent.	[60]
Observational study (n = 24; mean age = 9 years old)	Individual treatment with cannabis products for at least one year. THC doses ranged from 0.05 to 50 mg in 40% of children and CBD from 7.5 to 200 mg in 60% of children.	Among 65 potential cannabis-responsive biomarkers, 22 were categorized as anti-inflammatory, bioenergy associated, neurotransmitters, amino acids, and endocannabinoids. These biomarkers shifted toward typical physiological levels in children with ASD after cannabinoid treatment.	[61]
Randomized, double-blind, and placebo-controlled clinical trial (n = 60; mean age = 7 years old)	CBD-rich extracts at a concentration of 0.5%, in the ratio of 9CBD/1THC. The starting dose (2.5 mg/mL) was six drops, twice a day, with an increase of two drops daily twice a week, if necessary, up to a maximum dose of 70 drops daily.	CBD-rich extract treatment resulted in an improvement in social interaction, psychomotor agitation, number of meals, and anxiety. Concentration enhancement was only observed in mild ASD carriers. A total of 9.7% of the children experienced adverse effects that were mild and transient.	[62]

ADHD—attention-deficit/hyperactivity disorder; ADOS-2— Autism Diagnostic Observation Schedule 2; APSI—Autism Parenting Stress Index; CBD—cannabidiol; CBDV—cannabidivarin; CGI-I—Clinical Global Impression–Improvement scale; CSF—cerebrospinal fluid; CSHQ—Children’s Sleep Habit Questionnaire; HSQ-ASD—Home Situations Questionnaire for Autism Spectrum Disorder; PND—postnatal day; SRS—Social Responsiveness Scale; THC—tetrahydrocannabinol.

**Table 2:** Preclinical and clinical evidence on the use of cannabinoids in AD models and patients.

**Preclinical Studies**			
**Model**	**Treatment**	**Main Findings**	**References**
PC12 neuronal cells exposed to Aβ_1–42_ peptide	Pretreatment with CBD (10^−7^–10^−4^ M)	CBD prevented nitroxidative stress, apoptosis induction, tau protein hyperphosphorylation, and neurotoxicity induced by the Aβ_1–42_ peptide. Also, CBD inhibited iNOS via p38 MAPK and NF-κB.	[76,77,78]
AChE-induced Aβ_1–40_ peptide aggregation in vitro	THC (6.25–50 µM)	THC competitively inhibited AChE activity, as well as prevented AChE-induced Aβ peptide aggregation.	[79]
Intrahippocampal Aβ_1–42_ injection in mice	CBD (2.5 or 10 mg/kg, i.p.) for 7 days	CBD dose-dependently inhibited reactive gliosis and proinflammatory markers (iNOS and IL-1b) caused by the Aβ_1–42_ peptide.	[80]
Intrahippocampal Aβ_1–42_ injection in rats	CBD (10 mg/kg, i.p.) for 15 days	CBD interacted with PPARγ, reducing reactive gliosis and stimulating hippocampal neurogenesis.	[81]
Intracerebroventricular Aβ_1–40_ injection in mice	CBD (20 mg/kg, i.p.) daily for 7 days, following for 3 days/week for 2 weeks	CBD prevented learning deficit in the water maze task and IL-6 increased gene expression induced by Aβ_1–40_, but did not affect TNF-α gene expression.	[82]
SH-SY5Y neuroblastoma cells BV-2 microglia cells	THC and CBD (10 µM)	THC and CBD protected against microglial-activated neurotoxicity, but only CBD prevented Aβ neurotoxicity.	[83]
SH-SY5Y^APP+^ neuroblastoma cells	CBD (10^−7^–10^−4^ M)	Through PPARγ activation, CBD induced APP ubiquitination, resulting in decreased Aβ production and increased cell survival.	[84]
AβPP/PS1 mice aged 2.5 months	CBD (20 mg/kg, i.p.) daily for 3 weeks	CBD reversed social and novel object recognition memory impairments without affecting anxiety-related behaviors.	[85]
AβPP/PS1 mice aged 2.5 months	CBD (20 mg/kg, i.p.) daily for 8 months	CBD prevented the development of social recognition memory deficits, attenuated neuroinflammation, cholesterol, and dietary phytosterol retention, and did not impact anxiety, associative learning, Aβ load, or oxidative stress.	[86]
AβPP/PS1 mice aged 6 months (early symptomatic phase)	THC (0.75 mg/kg, i.p.); CBD (0.75 mg/kg, i.p.); THC + CBD (0.75 mg/kg, i.p., each) for 5 weeks	THC + CBD reduced learning impairment in object recognition and active avoidance tasks. Also, THC + CBD decreased soluble Aβ_1–42_ levels, changed plaque composition, and reduced astrogliosis, microgliosis, and proinflammatory molecules, more marked than with either THC or CBD.	[87]
APP/PS1 with CB2R knockout mice aged 3 and 6 months	THC + CBD (0.75 mg/kg, i.p., each) for 5 weeks	The CB2R absence exacerbated cortical Aβ deposition and soluble Aβ_1–40_ levels, without affecting the mice’s viability, tau hyperphosphorylation, memory impairment, and the positive cognitive effect demonstrated by THC + CBD.	[88]
AβPP/PS1 mice aged 12 months (advanced stages)	THC + CBD (0.75 mg/kg, i.p., each) for 5 weeks	THC + CBD was effective against memory impairment but did not modify Aβ processing or reduce glial reactivity. Mnemonic improvement was linked to reduced GluR2/3 and increased levels of GABA-A Rα1.	[89]
Hippocampal slices from C57BL6 mice exposed to Aβ_1–42_ peptide	CBD (10 µM)	Pretreatment with CBD restored the Aβ_1–42_-mediated deficit in LTP in the hippocampal CA1 region. The PPARγ antagonist blocked this effect.	[90]
MC65 neuronal cells exposed to Aβ HT22 hippocampal cells, primary neurons BV2 microglia cells	Cannabinoid compounds (250 nM–10 μM)	CBDA, THC, CBDV, Δ^8^-THC, CBG, CBC, CBN, and CBD removed intraneuronal Aβ, reduced oxidative damage, and offered protection from the loss of energy, trophic support withdrawal, and inflammation. THC and CBD lead to a synergistic neuroprotective effect.	[91]
TAU58/2 mice aged 4 months	CBD (50 mg/kg, i.p.) for 3 weeks	CBD did not affect the behavioral changes observed (anxiolytic like, motor impairment, and increased freezing in the fear conditioning paradigm).	[92]
Recombinant human tau protein 1N/4R isoform	Different CBD concentrations (0–40 μM)	CBD inhibits tau fibrils formation, by binding tau protein in Tyr residues. The tau–CBD complex formation is an exothermic process that occurs through hydrogen bonds and van der Waals forces in a spontaneous interaction.	[93]
Vero (CCL-81) pre-adipocytes (3T3-L1) (CL-173)	Cannabis extracts in hexane, DCM, DCM:MeOH (1:1), MeOH and water (1–100 μg/mL), CBD and THC (1–50 μg/mL), physostigmine (1–10 μg/mL)	Physostigmine and CBD inhibited AChE and BChE, while THC showed paltry inhibitory activity against both cholinesterases. Hexane, DCM, DCM:MeOH, MeOH, and water cannabis extracts did not inhibit AChE, but hexane and DCM cannabis extract inhibit BChE activity. All cannabis extracts inhibit β-secretase activity. Low cytotoxicity was observed.	[94]
Primary neurons, female ICR mice (8 weeks old) exposed to Aβ_1–42_	Primary neurons treated with Aβ_1–42_ and/or CBDA or THCA for 24 h, CBDA (6 μM, 3 μL/mouse), THCA (12 μM, 3 μL/mouse)	CBDA and THCA significantly suppressed neuronal cell death, ameliorated AD-like features by modulating Ca^2+^ levels, and reduced the APP increase, polymeric Aβ, oligomeric Aβ, and p-tau levels induced by Aβ_1–42._ CBDA or THCA induced a better performance in the Morris water maze and novel object recognition tests, reduced the increase in APP, polymeric Aβ, oligomeric Aβ, and p-tau levels induced by Aβ_1–42,_ and significantly increased BDNF, p-TrkB, and p-CREB levels.	[95]
**Clinical Studies**			
**Study Features**	**Treatment**	**Main Findings**	**Reference**
Randomized placebo-controlled double-blind crossover study (n = 12)	Placebo or THC 2.5 mg, p.o., for 6 weeks each	THC increased body weight and decreased agitation (CMAI), even during the placebo treatment period. Adverse reactions included euphoria, somnolence, and tiredness, without needed medication discontinuation.	[96]
Open label, prospective descriptive trial, single arm, single center (n = 6)	THC 2.5 mg, p.o., every evening for 2 weeks	THC reduced nocturnal motor activity and NPI score in 2 weeks, without significant impact on anxiety, apathy, delusions, hallucinations, or augmentative sedative medication use.	[97]
Single center randomized placebo-controlled double-blind crossover study (n = 2)	Placebo or THC 2.5 mg, p.o., every evening for 4 weeks each	THC initially decreased nocturnal motor activity, reduced NPI score after 4 weeks, and improved circadian rhythms (NPCRA) during treatment.	[98]
Retrospective cohort, single center (n = 40)	THC 7.03 mg/day for 4–7 days	THC decreased all domains of PAS, and improved CGI, sleep duration, and percentage of meals consumed during the treatment. There was no difference in GAF or change in weight. Sedation, delirium, urinary tract infection, and confusion were the most frequently documented adverse events. None of them led to medication discontinuation.	[99]
Multicenter randomized placebo-controlled double-blind crossover study (n = 50)	Placebo or THC 1.5 mg, p.o., 3×/day 3 weeks	No significant differences were found in the NPI score, CMAI, quality of life, activities of daily living according to the Barthel Index, vital signs, weight, episodic memory, and adverse effects between the placebo and THC.	[100]
Multicenter randomized placebo-controlled double-blind crossover study (n = 20)	Placebo and THC 0.75 mg, p.o., 2×/day, in random order, for 3 days, separated by a four-day washout for 6 weeks. After, the THC dosage was increased to 1.5 mg in the same design for 6 weeks.	THC did not reduce the NPI score compared to the placebo but was well tolerated, as assessed by adverse event monitoring, vital signs, and mobility.	[101]
Open label, placebo-controlled, prospective descriptive trial (n = 10)	Cannabis oil (THC 2.5 to a maximum of 7.5 mg) 2×/day for 4 weeks	Cannabis oil decreased the NPI score (decreased domains: delusions, agitation/aggression, irritability, apathy, sleep, and caregiver distress) and CGI in 4 weeks.	[102]
Randomized placebo-controlled double-blind crossover study (n = 18)	Placebo and THC 1.5 mg, p.o., 2×/day, in random order, for 3 days, separated by a 4-day washout.	THC elevated sway during standing eyes closed but not during standing eyes open, increased the stride length and trunk sway during preferred speed walking. No differences in the number and type of adverse events were found, and no falls occurred.	[103]
Case report (n = 1)	Cannabis extract (THC:CBD 8:1) (300 µg–1000 µg THC)	Microdoses of THC:CBD 8:1 improved MMSE and ADAS-Cog score. Cognitive enhancement was stable for more than one year. No significant side effects or toxicity were observed. The most effective dose for AD symptom suppression appeared to be 500 µg of THC.	[104]
Anecdotal, spontaneous, and observational study (n = 30)	Cannabis oil extract sublingually, twice daily for 12 weeks (1st–2nd week 15 drops/day; 3rd–4th week 23 drops/day; 5th–8th week 30 drops/day; 9th–10th week 23 drops/day; 11th–12th week 15 drops/day)	Reduction in typical AD behavioral problems, such as agitation, apathy, irritability, sleep disturbance, and eating disturbances, evaluated by the NPI questionnaire. Reduction in aggressive behavior, measured by the CMAI questionnaire. Overall, 45% of patients previously classified as severe cognitive decline were stratified as mild/moderate by the MMSE questionnaire. Positive feedback by the caregiver.	[105]

AChE—acetylcholinesterase, Aβ—amyloid beta peptide, ADAS-Cog—Alzheimer’s Disease Assessment Scale, APP—amyloid precursor protein, CBC—cannabichromene, CBD—cannabidiol, CBDA—cannabidiolic acid, CBDV—cannabidivarin, CBG—cannabigerol, CBN—cannabinol, CGI—Clinical Global Impression scores, CMAI—Cohen-Mansfield Agitation Inventory, DCM—dichloromethane, DMCBD—dimethyl cannabidiol, GABA-A Rα1—gamma-aminobutyric acid A receptors alpha 1 subunit, GAF—Global Assessment of Functioning, GluR2/3—glutamate receptor subunits 2 and 3, IL-1β—interleukin 1 beta, iNOS—inducible nitric oxide synthase, MeOH—methanol, MMSE—Mini-Mental State Examination, NF-κB—nuclear factor kappa B, NPCRA—Non-Parametric Circadian Rhythm Analysis, NPI—Neuropsychiatric Inventory, PAS—Pittsburgh Agitation Scale, p-p38 MAPK—phosphorylated P38 mitogen-activated protein kinases, PPARγ—peroxisome proliferator-activated receptor gamma, THC—tetrahydrocannabinol, TNF-α—tumor necrosis factor alpha.

## Data Availability

Not applicable.
